# Legionella-like and other amoebal pathogens as agents of community-acquired pneumonia.

**DOI:** 10.3201/eid0706.010619

**Published:** 2001

**Authors:** T J Marrie, D Raoult, B La Scola, R J Birtles, E de Carolis, Canadian Community-Acquired Pneumonia Study Group

**Affiliations:** Department of Medicine, University of Alberta, Edmonton, Canada. tom.marrie@ualberta.ca

## Abstract

We tested serum specimens from three groups of patients with pneumonia by indirect immunofluorescence against Legionella-like amoebal pathogens (LLAPs) 1-7, 9, 10, 12, 13; Parachlamydia acanthamoeba strains BN 9 and Hall's coccus; and Afipia felis. We found that LLAPs play a role (albeit an infrequent one) in community-acquired pneumonia, usually as a co-pathogen but sometimes as the sole identified pathogen.


### *Legionella*-Like and Other Amoebal Pathogens as Agents of Community-Acquired Pneumonia

**Thomas J. Marrie,* Didier Raoult,† Bernard La Scola,† Richard J. Birtles,† Emidio de Carolis,‡ and the Canadian Community-Acquired Pneumonia Study Group[1]**

*Department of Medicine University of Alberta, Edmonton, Alberta, Canada; †Faculté de Médecine de Marseille, Marseille, France; and ‡Pfizer Canada Inc., Montreal, Quebec, Canada

We tested serum specimens from three groups of patients with pneumonia by indirect immunofluorescence against *Legionella*-like amoebal pathogens (LLAPs) 1–7, 9, 10, 12, 13; *Parachlamydia acanthamoeba* strains BN 9 and Hall's coccus; and *Afipia felis*. We found that LLAPs play a role (albeit an infrequent one) in community-acquired pneumonia, usually as a co-pathogen but sometimes as the sole identified pathogen.

A number of bacteria that grow only within amoebae and are closely related phylogenetically to *Legionella* species, *Legionella*-like amoebal pathogens (LLAPs), have been identified and characterized (1). The role of these bacteria as human pathogens is still largely unknown. Other microorganisms, e.g., *Parachlamydia acanthamoeba* strains BN 9 (2) and Hall's coccus (3), also grow within amoebae. *Afipia felis* (once thought to be the etiologic agent of cat-scratch disease), a gram-negative rod, is difficult to grow on artificial medium but grows well in human monocytes and HeLa cells (4); this organism was recently reported to be an environmental bacterium probably associated with free-living amoebae and living in water (5). We tested serum specimens from three groups of patients with pneumonia to determine if any of these microorganisms cause disease.

### The Study

We used 511 specimens from a 1985 study of a random sample of the Nova Scotia population (6); 121 acute- and convalescent-phase serum specimens from a study (Nova Scotia, 1991-1994) of 149 ambulatory patients with community-acquired pneumonia (7); and specimens from a prospective study of community-acquired pneumonia requiring hospitalization conducted at 15 teaching hospitals in eight Canadian provinces (1996-1997).

All serum specimens from both groups of patients with pneumonia were tested for antibodies to *Mycoplasma pneumoniae*; influenza viruses A and B; parainfluenza viruses 1,2,3; adenovirus; and *Respiratory syncytial virus* (RSV) by a standard complement fixation technique in microtiter plates. Serum specimens from 60% of the patients (randomly selected from the group of patients with community-acquired pneumonia requiring hospitalization) were tested by the microimmunofluorescence test (8-10) for immunoglobulin (Ig) G and IgM antibodies to *Chlamydia pneumoniae* (AR 39 strain); *C. psittaci* (avian strain 6BC, feline pneumonitis strain FP, turkey strain TT 3, and pigeon strain CP 3); *C. pecorum* (ovine polyarthritis strain); and *C. trachomatis* (pooled antigens of serovars BED, CJHI, and FGK). Serum specimens from hospitalized patients with pneumonia were tested for antibodies to *Streptococcus pneumoniae* pneumolysin, pneumolysin immune complexes, C polysaccharide, surface protein A, *Haemophilus influenzae,* and *Branhamella catarrhalis* by Dr. M. Leinonen, National Public Health Institute, Oulu, Finland, as reported previously (11-13).

Acute- and convalescent-phase serum specimens from 150 patients also had been previously tested by enzyme-linked immunosorbent assay (ELISA) for antibodies to *L.pneumophila* serogroups 1–6 by Yu (14). A urine sample collected from each patient within 24 hours of hospitalization was tested for *L. pneumophila* serogroup 1 antigen by ELISA (15) (Binax, Inc., Portland, ME). Antibodies to *Coxiella burnetii* phase 1 and 11 antigens and to *Chlamydia pneumoniae* were determined by a microimmunofluorescence test, as described (16,17).

Antibody titers to LLAPs also were determined by the indirect fluorescent antibody technique. These included *Acanthamoeba polyphaga* strain Linc AP 1 and LLAP strains 1, 2, 4, 6, 7, 9, 10, 12; *L. lytica* (strains LLAP 3 and L2, formerly *Sarcobium lyticum*); and *Parachlamydia acanthamoeba* (strain BN 9 and Hall's coccus). *A. felis* ATCC 53690 was from the American Type Culture Collection. LLAPs were cultured in *A. polyphaga* in 150 peptone-yeast extract-glucose broth (18) at 30°C. When maximally infected, amoebae were lysed through three cycles of freeze-thawing in liquid nitrogen. This suspension was then resuspended in 30 mL of phosphate-buffered saline and centrifuged at 10,000 rpm for 10 minutes. Supernatant fluid was removed, and pellets containing respective LLAPs were resuspended in the smallest possible volume of sterile distilled water and

Address for correspondence: Thomas J. Marrie, Department of Medicine University of Alberta, 2F1.30 Walter C. Mackenzie Health Sciences Center, 8440 112th Street, Edmonton, Alberta, T6G 2R7 Canada; fax: 780-407-3132; e-mail: tom.marrie@ualberta.ca

[1]R. Duperval, S. Field, T. Louie, S. Houston, M. Gribble, K. Williams, L. Nicolle, R. Grossman, I. Salit, R. Saginur, D. Gregson, M. Laverdiere, Jean Joly, T. Marrie, and J. Hutchinson.

were adjusted to a concentration of 2 mg/mL, as determined spectrophotometrically. Antigen prepared in this manner was frozen at –20°C until required.

Specimens with an IgM titer of $\geq$1:100, seroconversion from 0 to 100, a fourfold rise in antibody titer between acute- and convalescent-phase serum, and a single or stable titer of $\geq$400 were considered indicative of recent infection. An antibody titer of $\geq$50 was considered seropositive, i.e., evidence of prior but not recent infection.

The seropositivity rate to various antigens is shown in Table 1. The background rate of such infection is low. Only one healthy Nova Scotian had serologic evidence of recent infection with an LLAP, LLAP 4. None of the patients with ambulatory pneumonia had such an infection. LLAP 4 was the most common LLAP-causing pneumonia: four such infections among patients with community-acquired pneumonia required hospitalization. Two of the 58 patients from the Nova Scotia site had infection with LLAP 4, versus one of 511 healthy Nova Scotians (p<0.029, Fisher exact test). BN 9 caused two infections, and LLAP 1 and 12 caused one each among patients with community-acquired pneumonia requiring hospitalization.

### Case Histories

### LLAP 1 and 12 Infections

A 40-year-old floral designer was hospitalized in Edmonton, Alberta, with a 21-day history of diarrhea, myalgias, headache, chills, and shortness of breath. She had traveled to Los Angeles and Palm Springs in the previous 10 days. On admission, her oral temperature was 36.9°C, and fine bilateral interstitial infiltrates were present on a chest radiograph. She was treated with erythromycin and doxycycline, was discharged on day 7, and was readmitted 2 weeks later, at which time a transbronchial biopsy specimen yielded *Mycobacterium avium intracellulare* on culture. No evidence of HIV infection was found. The acute- and convalescent-phase antibody titers to LLAP 1 were 1:400 in the IgG fraction and 1:25 and 0 in the IgM fraction.

A 34-year-old clerical worker in a hospital radiology department was hospitalized on April 11, 1996, with pleuritic chest pain and shortness of breath of 10 days' duration. Her oral temperature was 38.7°C. The leukocyte count was 9.2 x $10^9$ /L. A chest radiograph showed multilobar patchy opacities on the left and a 3-cm nodular opacity on the right. The patient was treated with erythromycin and cefuroxime intravenously for 36 hours, followed by oral clarithromycin. The nodule did not resolve over the next 6 weeks, and an open lung biopsy was performed. All cultures were negative. Histologic examination revealed acute and chronic inflammation. The acute-phase serum sample had an IgM antibody titer of 1:200 to LLAP 12, and the convalescent-phase titer was 1:100; the corresponding values for IgG were 0 and 1:50.

Table1.Percent seropositivity (antibody titer $\geq$1:50) to various antigens among three study groups[a]

| Antigen | Healthy Nova Scotians (%) N = 511 | Ambulatory pneumonia (%) N = 121 | CAP requiring hospitalization (%) N = 255 |
|---|---|---|---|
| LLAP-1 | 0.19 | 1.6 | 0.7 |
| LLAP–2 | 0 | 0 | 0.39 |
| LLAP–3 | 0.39 | 1.6 | 0.7 |
| LLAP-4 | 0.39 | 0 | 4.3 |
| LLAP–6 | 0.1 | 0 | 0.39 |
| LLAP-7 | 1.36 | 0 | 1.56 |
| LLAP–9 | 0.39 | 1.6 | 0.7 |
| LLAP-10 | 0 | 0 | 0.7 |
| LLAP-12 | 0.97 | 1.6 | 0.39 |
| Hall's coccus | 0 | 1.6 | 2.35 |
| BN 9 | 0 | 0 | 2.35 |
| *Afipia felis* | 0 | 0.82 | 0 |

[a]As defined in paper.

CAP = community-acquired pneumonia.

### BN 9 Infection

A 21-year-old university student was hospitalized with fever, abdominal pain, nausea, vomiting, diarrhea, pleuritic chest pain, and nonproductive cough. He also complained of a sore throat and shortness of breath. On examination, he looked acutely ill and had a diffuse erythematous rash. His oral temperature was 38.3°C. A chest radiograph showed diffuse opacities involving both lower lobes. He was treated with erythromycin. The next day desquamation of the lips and the skin of the digits was noted, and a diagnosis of adult Kawasaki disease was entertained. Treatment with aspirin and gamma globulin was instituted, and the patient made an uneventful recovery. There was no evidence of cardiac involvement as indicated by normal serial electrocardiograms and a normal echocardiogram. The BN 9 antibody titer was 1:50 and 1:6,400 in the acute- and convalescent-phase serum specimens. There was a stable antibody titer to Hall's coccus of 1:400 in both. Blood and urine cultures, as well as other microbiologic tests were negative.

A 68-year-old man was hospitalized on October 15, 1996, with nausea, vomiting, diarrhea, a nonproductive cough, shortness of breath, chills, and pleuritic chest pain. The year before, he had received a cadaveric renal transplant and was maintained on corticosteroid and cyclosporin therapy. His oral temperature was 39.2°C, and consolidation was found on examination of the right lung. A chest radiograph showed a single lobar opacity on the right. The leukocyte count was 17 x $10^9$/L. *S. pneumoniae* was isolated from the sputum. The patient was treated with cefuroxime intravenously for 4 days and was discharged on oral cefaclor. He made an uneventful recovery. The acute- and convalescent-phase titers to BN 9 were stable at 1:400.

### LLAP 4 Infection

Four patients met our definition for infection with LLAP 4 (Table 2). Appearance of pneumonia was similar in all four chest radiographs. Patient ML 13 had diffuse interstitial infiltrates, but this patient, who had had a bone marrow transplant, also had RSV infection. All patients with LLAP 4 recovered from pneumonia.

### Conclusions

In August 1986, Rowbotham (19) isolated LLAP 1 from the sputum of an 82-year-old woman with persistent

Table 2. Summary of selected characteristics of four patients infected with *Legionella*-like amoebal pathogen (LLAP)–4

| No. | Age | Sex | Temp | Symptoms | LOS | WBC | Antibiotics | Comments | Antibody titer | Co-pathogens |
|---|---|---|---|---|---|---|---|---|---|---|
| TM37 | 93 | M | 38.3 | nausea, myalgia, shortness of breath, nonproductive cough, chest pain | 8 | 18.2 | erythromycin; cefuroxime | admitted from a nursing home, sustained a non q wave myocardial infarction | IgG 1:400 and 1:400 | None |
| TM11 | 87 | F | 39.0 | nonproductive cough, chills | 11 | 8.7 | erythromycin; cefuroxime | | IgG 0 and 1:100 | None |
| ML13 | 54 | M | 38.4 | abdominal pain, nonproductive cough, myalgia, chest pain | 17 | 2.2 | erythromycin; ceftazidime, ribavirin | bone marrow transplant; required intensive care unit treatment | IgM 25 and 0; IgG 200 and 1:400 | RSV |
| LN 9 | 63 | M | 36.8 | shortness of breath, chills | 10 | 20.8 | erythromycin, cefuroxime | | IgM 1:200 and 1:200 | *Strepto-coccus pneumoniae* |

M = male; F = female; LOS = length of stay in days; wbc = leukocyte count; antibody titer = first value is from acute-phase sample and second from convalescent-phase sample.

pneumonia, by cocultivation with *A. polyphaga*. Seroconversion was demonstrated to LLAP 3. He screened >5,000 serum specimens submitted for *Legionella* antibody testing and found that 10 patients met the criteria for infection with LLAP 3 (19).

The only other study similar to ours is a study by Benson et al. (20), who examined 500 patients with community-acquired pneumonia and determined antibody titers to LLAP 1,2,3,4,6,7,9, and Hall's coccus; 94 (18.9%) had a fourfold rise in antibody titer of ≥128 to any LLAP; 36 (7.2%) had a titer rise to ≥1,024. In contrast, 1.4% of our 255 hospitalized patients with community-acquired pneumonia had evidence of recent infection with a LLAP or Hall's coccus. As in our series, LLAP 4 was the most common cause of infection in the Benson study, which also found that in 10 (10.6%) of 94 patients with LLAP or Hall's coccus infection a copathogen had been implicated as cause of the pneumonia. Likewise, almost all our patients with LLAP infection were infected with another pathogen.

One of the most interesting findings in our study was a fourfold rise in antibody titer to BN 19 in a patient with presumed adult-onset Kawasaki syndrome, an acute vasculitis of unknown cause found predominantly in infants and young children. The diagnostic criteria include fever of >5 days plus four of the following five features: bilateral conjunctivitis without exudate; polymorphous eruption; cervical lymph node >1.5 cm in diameter; changes in the extremities, including edema of the hands or feet, palm or sole erythema, and periungual desquamation during convalescence; and changes in the oropharynx, including fissured red lips, strawberry tongue, and diffuse erythema of the oropharyngeal mucosa (21). Our patient met this definition. An association between an antecedent respiratory infection and Kawasaki syndrome has been described (21,22), as has exposure to freshly cleaned carpets (23,24). It is possible that the gamma globulin administered to our patient contained antibody to BN 19. However, there was no seroconversion or high titer of antibody to any of the other antigens included in our test panel. A possible association between infection with BN 19 and Kawasaki syndrome is easily tested.

Strengths of this study are its size and the comprehensiveness of the diagnostic work-up. Its limitations include the following: the three populations were enrolled in different periods; we tested only a subset of the patients hospitalized with community-acquired pneumonia and these patients were from multiple centers across Canada; our comparison groups (healthy persons and patients with ambulatory pneumonia) were Nova Scotians. However, the inferences that we are making are limited to the rate of infection in these three separate groups and are not intended to indicate differences temporally or geographically.

Our data suggest that LLAPs play a role, albeit an infrequent one, in community-acquired pneumonia. Usually they are a copathogen, but in some cases they are the sole pathogen. The possible association between BN 9 and Kawasaki disease requires further study.

### Acknowledgments

We thank the following study coordinators: M. Dumbreville, H. Salts, J. Clark-DiPrata, B. Peters, K. Henery, J. Graham, F. Brisebois, H. Patil, G. Patrick, T. Muir, F. Hebel, E. Condon, S. Roberts, A. Lindemudder, D. Piget-Dellio, M. Jones, and C. Hammerberg. *Parachlamydia acanthamoeba* strains VN 19 and Hall's coccus were kindly provided by R.J. Birtles and T.J. Rowbotham.

Funding was provided by Pfizer Canada Inc., Pfizer United States (urinary antigen testing, courtesy of Dr. Jill Inverso) and Laboratory Centre for Disease Control, Winnipeg, Manitoba (*C. pneumoniae* testing).

Dr. Marrie is professor and chair of the Department of Medicine at University of Alberta. His primary research interest is community-acquired pneumonia.

### References

1. Birtles RJ, Rowbotham TJ, Raoult D, Harrison TG. Phylogenetic diversity of intra-amoebal legionellae as revealed by 16S rRNA gene sequence comparison. Microbiology 1996;142:3525–30.

2. Everett KDE, Bush RM, Andersen AA. Emended description of the order *Chlamydiales*, proposal of *Parachlamydiaceae* fam. nov. and *Simkaniaceae* fam. nov., each containing one monotypic genus, revised taxonomy of the family *Chlamydiaceae*, including a new genus and five new species, and standards for the identification of the organisms. Int J Syst Bacteriol 1999;49:415–40.
3. Lewis DM, Dutkiewicz J, Sorenson WG, Mamolen M, Hall JE. Microbiological and serological studies of an outbreak of humidifier fever in a print shop. Biodeterioration Research 1990;3:467-77.
4. Birkness KA, George VG, White EH, Stephens DS, Quinn FD. Intracellular growth of *Afipia felis*, a putative etiologic agent of cat scratch disease. Infect Immun 1992;60:2281–7.
5. La Scola B, Raoult D. *Afipia felis* in hospital water supply in association with free living amoebae. Lancet 1999;353:1330.
6. Marrie TJ, Pollak PT. Seroepidemiology of Q fever in Nova Scotia: evidence for age dependant cohorts and geographical distribution. Eur J Epidemiol 1995;11:47-54.
7. Marrie TJ, Peeling RW, Fine MJ, Singer DE, Coley CM, Kapoor WN. Ambulatory patients with community-acquired pneumonia: the frequency of atypical agents and clinical course. Am J Med 1996;101:508–15.
8. Grayston JT, Wang SP, Kuo C-C, Campbell LA. Current knowledge of *Chlamydia pneumoniae*, strain TWAR, an important cause of pneumonia and other acute respiratory diseases. Eur J Clin Microbiol Infect Dis 1989;8:191-202.
9. Fukushi H, Hlrai K. Proposal of *Chlamydia pecorum* sp. nov. for Chlamydia strains derived from ruminants. Int J Syst Bacteriol 1992;42:306–8.
10. Wang S-P, Kuo C-C, Grayston JT. Formalized *Chlamydia trachomatis* organisms as antigens in the microimmunofluorescence test. J Clin Microbiol 1979;10:259–61.
11. Jalonen E, Taira S, Paton JC, Kerttula Y, Suomalainen P, Leinonen M. Pneumolysin produced in *Bacillus subtilis* as antigen for measurement of pneumococcal antibodies by enzyme immunoassay. Serodiagnosis and Immunotherapy of Infectious Diseases 1990;4:451-8.
12. Jalonen E, Paton JC, Koskela M, Kerrtula Y, Leinonen M. Measurement of antibody responses to pneumolysin—a promising method for the presumptive aetiological diagnosis of pneumococcal pneumonia. J Infect 1989;19:127–34.
13. Leinonen M, Syrjala H, Jalonen E, Kujala P, Herva E. Demonstration of pneumolysin antibodies in dissociated immune complexes—a new method for etiological diagnosis of pneumococcal pneumonia. Serodiagnosis and Immunotherapy of Infectious Diseases 1990;4:459–68.
14. Elder EM, Brown A, Remington JS, Naot Y. Microenzyme-linked immunoabsorbent assay for detection of immunoglobulin G and immunoglobulin M antibodies to *Legionella pneumophila* . J Clin Microbiol 1983;17:112–21.
15. Berdal BP, Farshy CE, Feeley JC. Detection of *Legionella pneumophila* antigen in urine by enzyme-linked-immunospecific assay. J Clin Microbiol 1979;9:575-8.
16. Marrie TJ, Van Buren J, Faulkner RS, Haldane EV, Williams JC, Kwan C. Seroepidemiology of Q fever in Nova Scotia and Prince Edward Island. Can J Microbiol 1984;30:129–34.
17. Schachter J, Dawson CR. Human chlamydial infections. Littleton (MA): PSG Publishing Co. Inc.; 1978. p. 24.
18. Rowbotham TJ. Isolation of *Legionella pneumophila* from clinical specimens via amoebae and the interaction with those and other isolates with amoebae. J Clin Pathol 1983;36:978-86.
19. Rowbotham TJ. *Legionella*–like amoebal pathogens. In: Barbaree JM, Breiman RF, Dufour AP, editors. Legionella–current status and emerging perspectives. Washington: American Society for Microbiology; 1993. p. 137-40.
20. Benson RF, Drozanski WJ, Rowbatham TJ, Bialkowska I, Losos D, Butler JC, et al. Serologic evidence of infection with 9 Legionella-like amoebal pathogens in pneumonia patients. Proceedings of the 95th ASM General Meeting; 1995 May 21-25; Washington, DC, USA. [Abstract C-200. p. 35.]
21. Bell DM, Brink EW, Nitzkin JL, Wulff H, Berkowitz ID, Feorino PM, et al. Kawasaki syndrome: description of two outbreaks in the United States. N Engl J Med 1981;304:1568-75.
22. Dean AG, Melish ME, Hicks R, White ME. An epidemic of Kawasaki syndrome in Hawaii. J Pediatr 1982;100:552–7.
23. Patriarca P, Rogers M, Morens D, Schonberger LB, Kaminski RM, Burns JC, et al. Kawasaki syndrome: association with appliation of rug shampoo. Lancet 1982;2:578–80.
24. Rogers M, Kochel R, Hurwitz E, Jillson CA, Hanrahen JP, Schoenberger LB. Kawasaki syndrome: is exposure to rug shampoo important? Am J Dis Child 1985;139:777–9.
